# Photo-Induced Active Lewis Acid–Base Pairs in a Metal–Organic Framework for H_2_ Activation

**DOI:** 10.1021/jacs.3c05244

**Published:** 2023-08-24

**Authors:** Bryan Kit Yue Ng, Zi-Jian Zhou, Ting-Ting Liu, Tatchamapan Yoskamtorn, Guangchao Li, Tai-Sing Wu, Yun-Liang Soo, Xin-Ping Wu, Shik Chi Edman Tsang

**Affiliations:** † Department of Chemistry, University of Oxford, Oxford OX1 3QR, U.K.; ‡ Key Laboratory for Advanced Materials, Centre for Computational Chemistry and Research Institute of Industrial Catalysis, East China University of Science and Technology, Shanghai 200237, People’s Republic of China; ∥ National Synchrotron Radiation Research Center, 101 Hsin-Ann Road, Hsinchu 30076, Taiwan; ⊥ Department of Physics, National Tsing Hua University, Hsin-chu 30013, Taiwan

## Abstract

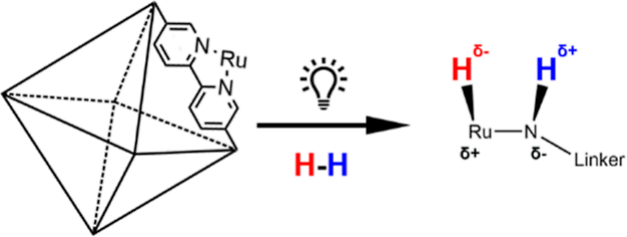

The establishment of active sites as the frustrated Lewis pair (FLP) has recently attracted much attention ranging from homogeneous to heterogeneous systems in the field of catalysis. Their unquenched reactivity of Lewis acid and base pairs in close proximity that are unable to form stable adducts has been shown to activate small molecules such as dihydrogen heterolytically. Herein, we show that grafted Ru metal–organic framework-based catalysts prepared via N-containing linkers are rather catalytically inactive for H_2_ activation despite the application of elevated temperatures. However, upon light illumination, charge polarization of the anchored Ru bipyridine complex can form a transient Lewis acid–base pair, Ru^+^–N^–^ via metal-to-ligand charge transfer, as confirmed by time-dependent density functional theory (TDDFT) calculations to carry out effective H_2_–D_2_ exchange. FTIR and 2-D NMR endorse the formation of such reactive intermediate(s) upon light irradiation.

## Introduction

The activation of H–H bond is challenging in various catalytic process for the production of fuels.^1^ Traditional oxidative addition steps over an electron–rich transition metal center can lead to H–H cleavage into two M–H bonds. Recently, frustrated Lewis pairs (FLPs) involving synergetic sites can be created from stereoisolated Lewis acid and base sites in close proximity or derived from a weak intramolecular adduct instead of using single transition metal center to provide an alternative pathway for the heterolytic H–H bond cleavage by the charged, polarized bodies. From the pioneering work of Welch and Stephan, who reported the heterolytic fission of the H–H bond by sterically bulky phosphines and boranes,^2^ the application of FLP in catalysis have been prominent in the activation of different small molecules including CO_2_ and alkynes since the extension of the concept into heterogeneous systems by combining imines and nitriles as Lewis base and the gold surface as Lewis acid.^3^ Geometrically separated anion vacancies and hydroxyl groups have then been reported as Lewis basic and acidic sites, respectively.^4–6^ However, the majority of such FLP catalysts have the Lewis basic and acidic sites separately introduced, which prohibits precise control of the location and quantity of the active sites. Further advancement in synthesis methods would be required for the improved catalytic application of FLP for small-molecule activation by enhanced control of the active sites.

Molecular activation via photochemical means is generally achieved under comparatively milder conditions. Photocatalytic activation of the H–H bond would therefore be beneficial toward energy efficiency. Our approach for the design of such photoactive transient but polarized Lewis acid–base sites in metal–organic framework (MOF) materials are inspired by the characteristic superior surface area and porosity of the MOFs and their anchored transition metal(s) with designated linkers of tunable properties to capture light energy with facilitated polarization, which could create the required active Lewis acid and base pair for synergetic activation of substrate.^7^ In our previous work, we have utilized various MOFs as supports for the design of efficient catalysts thanks to their vast chemical tunability.^8^ MOFs are extending frameworks formed by the coordination of metal clusters with polyfunctional organic ligands, specifically the Universitet-i-Oslo (UiO) series.^9^ Its incredible chemical stability is credited to the strong electrostatic attraction between highly charged Zr_6_O_4_(OH)_4_ clusters and carboxylic acid-based organic linkers.^10–12^ Its aforementioned adjustable functional groups and tunable pore sizes^13–15^ have attracted considerable attention for applications in adsorption,^16^ separation,^17^ sensing,^18,19^ and catalysis.^20–22^ Thanks to such tunability of the UiO series, the ruthenium bipyridine motives can be integrated into the MOF systems. Ruthenium has been deposited onto MOF UiO-67-bpydc with 2,2′-bipyridine-5,5′-dicarboxylic acid analogous to ruthenium bipyridine complexes^23,24^ (denoted Ru/bpy). For comparison, ruthenium–amine complexes were also synthesized with ruthenium-decorated UiO-66-NH_2_ (denoted Ru/NH_2_), where 2-aminoterephthaclic acid was used as the organic linker.

In this work, we demonstrate the high activity for H_2_–D_2_ exchange of our photoinduced FLP-like catalyst with ruthenium deposited UiO-67-bpydc (Ru/bpy) under illumination. Our synthesis method gives precise control of the FLP-like active site location and quantity in the solid catalyst. Synchrotron X-ray absorption and X-ray diffraction measurements were initially performed to determine the structures of the anchored ruthenium complexes on the frameworks with precision before theoretical calculations. The optical properties of different Ru-MOFs were also characterized with UV–vis, PL, and TRPL. Excited-state calculations based on time-dependent density functional theory (TDDFT) were particularly performed to rationalize the charge transfer processes and the formation of photoinduced active sites in excited states. IR and 2D NMR indicated the synergetic formation of hydride and proton from dihydrogen via the formation of a transient Lewis acid–base pair upon illumination.

## Results and Discussion

**Figure 1:**
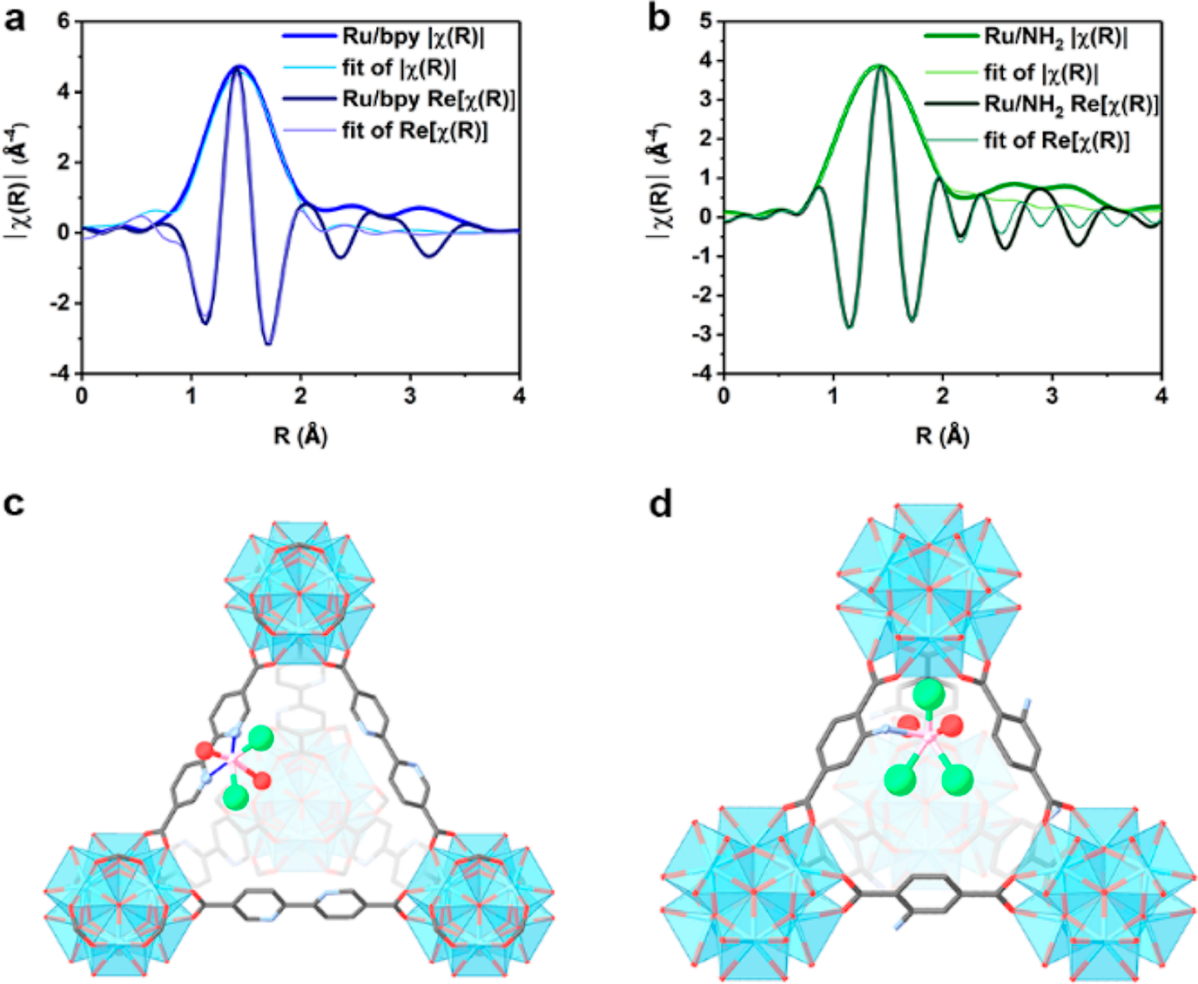
Fourier-transformed magnitude of the experimental Ru K-edge *k*^3^-weighted *R*-space EXAFS data and fit of (a) Ru/bpy and (b) Ru/NH_2_, where |χ(*R*)| and Re[χ(*R*)] denote the magnitude and real part of the Fourier transformed *k*-space data. Crystal structures built from the EXAFS fitting parameters of (c) Ru/bpy and (d) Ru/NH_2_. Color scheme: Zr = light blue polyhedra, O = red, C = black, N = blue, Ru = pink, and Cl = light green. Hydrogen and adsorbed water O atoms have been omitted for clarity.

Ruthenium-deposited UiO-67-bpydc (bipyridine linker) and UiO-66-NH_2_ (benzylamine linker) were synthesized as prototypes for photoinduced FLP. Inductively coupled plasma-mass spectrometry (ICP-MS) results on both Ru/bpy and Ru/NH_2_ showed similar Ru/Zr ratios of 0.352 and 0.344 (Table S1). Advanced techniques were then utilized to characterize the structures of their Ru loaded samples, namely, Ru/bpy (UiO-67-bpydc with Ru) and Ru/NH_2_ (UiO-66-NH_2_ with Ru). First, extended X-ray absorption fine structure (EXAFS) was performed on the two ruthenium-deposited MOFs to probe their local coordination environment. The scattering paths generated from bulk RuN, RuO_2_, and RuCl_3_ were then used to fit the *R*-space EXAFS data (Figure 1a,b), with the *k*-space data and fit available in Figure S1 and fitting parameters available in Table S2. The goodness of fit in the EXAFS data is shown by the low *R*-factor of 0.59 and 0.04% for Ru/bpy and Ru/NH_2_, respectively. In both Ru/NH_2_ and Ru/bpy, it is unambiguous that there is no aggregation of the ruthenium species in the system by comparing the data to the scattering paths generated from the bulk references RuN, RuO_2_, RuCl_3_, and Ru foil (Figure S2). In total, an average of ca. 6 coordination 6.4(7) for Ru/bpy and 5.8(5) for Ru/NH_2_ can be fitted. The degeneracy obtained from fitting for Ru/bpy shows a value of 2.0(2) Ru–N from the bipyridine linker, 2.2(3) Ru–O from adsorbed H_2_O and 2.2(2) Ru–Cl. For Ru/NH_2_, 0.9(1) Ru–N from linker, 1.9(1) Ru–O, and 3.0(3) Ru–Cl. To further determine their structures, synchrotron X-ray diffraction (SXRD) was performed on all the samples and is shown in Figure S3. Notice that the positions of the Bragg peaks remain unchanged (space group: *Fm*3̅*m*) for both samples, implying that the crystalline framework of the host MOF remains mostly unaltered by the Ru incorporation. Further Rietveld refinement of the model was built based on the bond lengths from EXAFS fitting resulted to generate an SXRD pattern and a satisfactory fit (*R*_wp_ values of 15.46 and 10.58 for Ru/bpy and Ru/NH_2_, respectively) with acceptable parameters was obtained through the Rietveld method.^25^ The refined structures are shown in Figure 1c,d, with the Rietveld refined parameters and fit in Table S3.

## Structure–Activity Relationship

**Figure 2:**
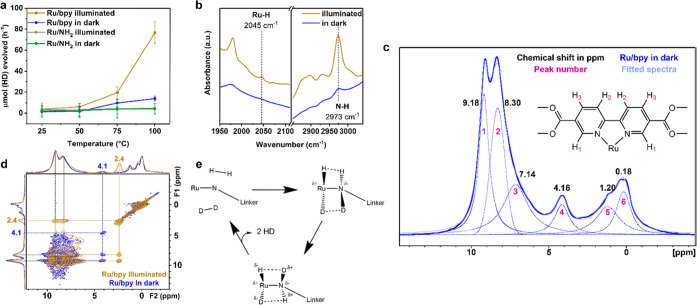
(a) Amount of HD formed with both catalysts, with and without light. (b) FTIR spectra of Ru/bpy in the dark and illuminated in reaction conditions. (c) Deconvoluted ^1^D ^1^H SSNMR spectra of Ru/bpy in the dark. (d) ^1^H–^1^H COSY NMR spectrum of Ru/bpy in the dark (blue) and illuminated (brown). (e) Proposed mechanism of heterolytic activation of the H–H bond by transient frustrated Lewis acid–base like pair.

The hydrogen activation of Ru/bpy and Ru/NH_2_ was evaluated by hydrogen–deuterium exchange. Reactions with and without visible light filtered Xe lamp irradiation were performed at 25 to 100 °C, and the results are presented in Figure 2a. The conversion factor of the signal of the quadruple mass spectrometer was determined by a calibration curve of pure HD gas (Figure S4). There is no noticeable exchange rate with both the Ru/bpy and Ru/NH_2_ samples at room temperature. At 100 °C, small thermal activation of about 10 μmol HD per hour is formed and can be detected over Ru/bpy due to a thermal partition of the electrons for metal-to-ligand charge transfer to a small degree. Conversely, Ru/NH_2_ still does not seem to show any measurable activity in the dark. However, under illumination, there is a dramatic light promotion to the exchange rate with at least ca. 5.5-fold increase in the amount of HD formed in Ru/bpy, while Ru/NH_2_ still remains catalytically inert. The turnover frequency, with the Ru content based on ICP-MS results, is plotted against inverse of temperature to obtain an activation energy of 0.368 eV under light illumination (Figure S5), attributed to the diffusion of H_2_/D_2_ molecules into the pores of Ru/bpy. This is much lower than the literature value of 0.672 eV for thermal chemical exchange between H_2_ and D_2_, and this reduction is associated with the Ru–N catalyzing the reaction in an FLP-like manner.^26^ It is apparent that the two Ru-MOF samples do not seem to be active for H_2_ activation via dissociative means over their metal center at room temperature. In fact, the more extensive conjugated aromatic π* in the bipyridine in Ru/bpy should in principle be able to withdraw electron from Ru by the back-donation than the electron richer Ru from the sigma Ru–N bond in Ru/NH_2_, hence attenuating the propensity for classical H_2_ activation. However, this catalyst is clearly shown to be more active under light at elevated temperature than the Ru/NH_2_. The key question is why H_2_ activation can be significantly promoted by light in the Ru/bpy but it does not apply to the related Ru/NH_2_.

To understand the mechanistic pathway of the H_2_–D_2_ exchange reaction, Fourier transformed infrared (FTIR) spectroscopy at variable temperature was performed on the reaction intermediate (Figures 2b and S6). A simultaneous detection of Ru–H and N–H stretching at 2045 cm^–1^ of Ru–H and at 2973 cm^–1^ of N–H in the quenched sample at 100 °C supporting the unconventional heterolytic cleavage of H–H upon illumination is identified.^27,28^ Despite the difficulty in quantification by infrared, the Ru–H appears to be far smaller in size than that of N–H. It is well-known that charge transfer and proton migration can take place simultaneously from transition metal hydride with a proton acceptor in close proximity. For example, [FeFe]-hydrogenases can catalyze H_2_ oxidation to protons exclusively via initiative FLP Fe–N sites and with the further conversion of H^–^ on Fe by charge transfer to NH^+^. Similar chemistry for Ru–H charge transfer and proton migration to O(H^+^) on MgO(111) is demonstrated.^29^ Thus, Ru^3+^ (2N) can generate Ru^+^ and 2NH^+^ with a substantial reduction/elimination of the Ru–H peak. Notice that one-dimensional (1D) ^1^H solid-state nuclear magnetic resonance (SSNMR) spectra for sample before and after illumination are shown in Figure S7. Deconvolution of the spectrum of Ru/bpy in dark shows a 1:0.87:0.91 ratio of the peaks at 9.18, 8.30, and 7.14 ppm, respectively, of the three H environments on the linker, as depicted in Figure 2c and Table S4.^30,31^ The resonances at around 1.20 and 0.18 ppm are attributed to the bridging Zr(μ_3_-OH) groups^32^ and the linker defects motifs,^33^ respectively. Interestingly, a peak at 4.1 ppm, characteristic of trapped H_2_ in the porous sample, is detected before light activation. For well-flushed and cold-trapped sample after illumination, its NMR spectrum is also deconvoluted and shown in Figure S8 and Table S5. Although no similar trace peak of Ru–H as IR is identified, presumably embedded in strong background, a distinctive peak at 2.4 ppm attributable to proton on amine can clearly be seen upon the illumination.^34^ The relative proton peak size of bipyridine (7–9 ppm) to NH^+^ (2.4 ppm) peak of 7.19:1 suggests the near conversion, with the stoichiometric quantity of Ru–N pairs relative to bipyridinic protons determined by ICP and thermogravimetric analysis (TGA, Figure S9 and Tables S6 and S7) as 6.90:1. To correlate their spatial relationship, a two-dimensional (2D) ^1^H–^1^H magic angle spinning (MAS) SSNMR experiment was performed (Figure 2c). Besides, the strong cross-diagonal correlation peaks which belong to the equivalent proton sites, both peaks at 4.1 and 2.4 ppm show off-diagonal correlation peak with the bipyridinic protons, indicating their spatial close proximity. Evidently, transient N(H^+^) can be identified in H_2_ with no reduction to the bipyridine linker during illumination (proton remained in the same positions) after activation and charge transfer characterized by IR and ssNMR. It is thought that light activation is likely to create transient intramolecular Ru^+^–N^–^ via metal-to-ligand charge transfer (MLCT) for H_2_ activation. We therefore propose a photoinduced Ru–N polarization mechanism as depicted in Figure 2e. H_2_ and D_2_ molecules can be exchanged by light activation.

## Optical Characterization

**Figure 3:**
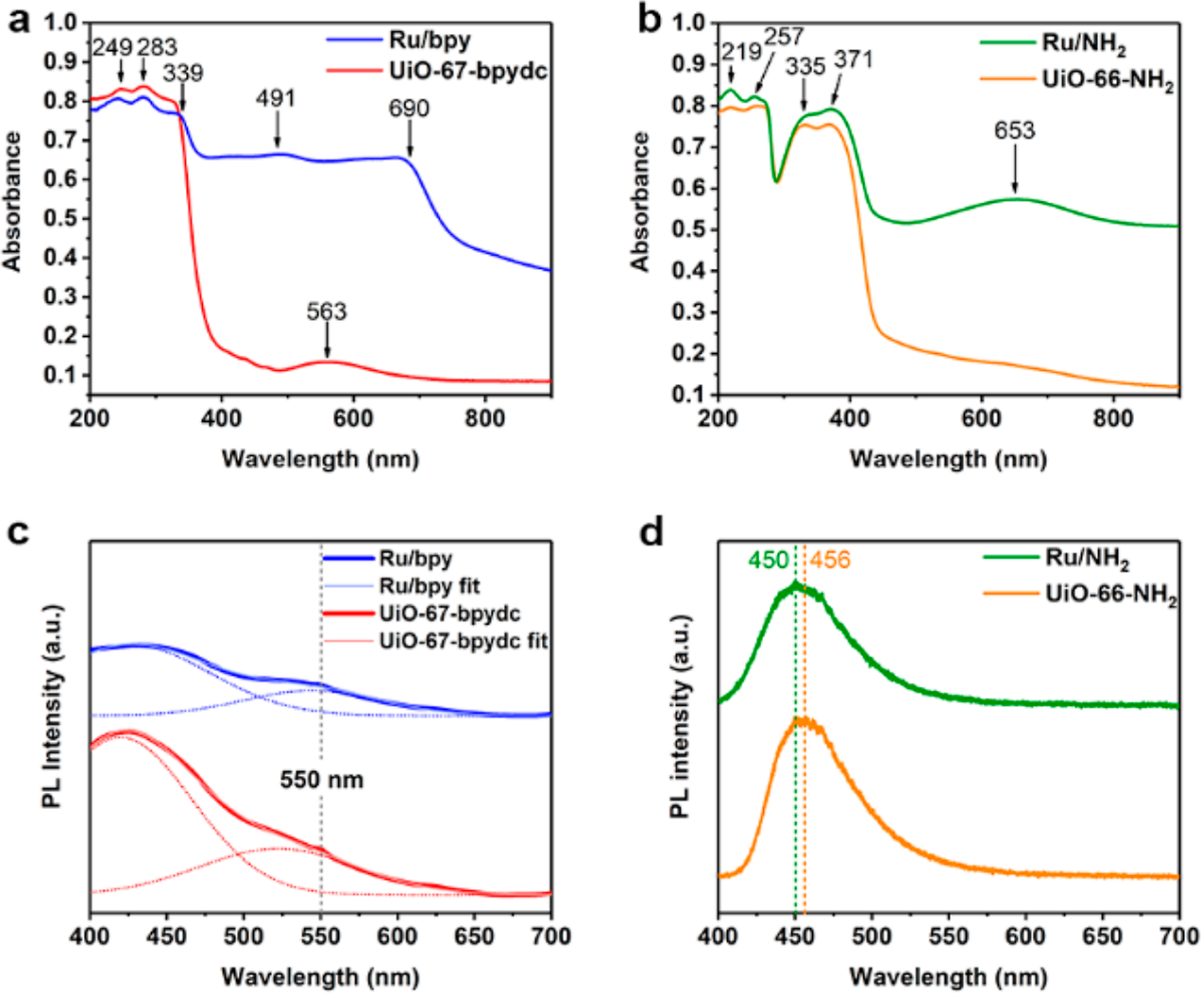
UV–vis absorbance spectra comparison for (a) Ru/bpy, UiO-67-bpydc, and (b) Ru/NH_2_, UiO-66-NH_2_. Photoluminescence spectra of (c) Ru/bpy and (d) Ru/NH_2_ along with their parent MOFs were excited at 375 nm.

To confirm the importance of MLCT over Ru/bpy over Ru/NH_2_ to create the active Lewis acid–base pair, the various charge transfer processes of different energies of the ruthenium-decorated MOFs were investigated by UV–vis spectroscopy (Figure 3a,b). Observed in both UiO-67-bpydc and Ru/bpy, the absorption peaks at 249 and 283 nm can be attributed the typical π to π* transition of a bipyridinic and the isolated pyridinic rings present in these MOF samples, respectively.^35^ For the absorption characteristics at visible regime, UiO-67-bpydc without Ru, gives a characteristic peak at 563 nm which can be ascribed to linker–linker charge transfer (LLCT), as it is also detectable in its free linker molecules (Figure S10). Interestingly, three distinctive intense broad peaks commonly assigned as MLCT peaks are indeed detectable in Ru/bpy around 339, 491, and 690 nm.^36–38^ Such strong adsorption peaks cover almost the entire visible region, giving the intense dark color of the sample. On the other hand, in the case of benzyl amine linker (UiO-66-NH_2_) with the linker of lesser conjugation, the π to π* absorption peaks are found to be located at 219 and 257 nm, respectively.^35,39^ Additional peaks at 335 and 371 nm can be generally ascribed to the charge transfer processes from amine lone pair to organic linker π* transitions,^40^ which remain unaltered upon Ru incorporation. With rather isolated conjugated benzyl ring from the sigma M–NH_2_ there is no similar broad intense LLCT or MLCT absorption regions at the visible region in both samples in contrast to those observed in bipyridine systems. Only a small peak around 653 nm of Ru/NH_2_ is detected, which may be attributed to the weak Ru d–d transitions.^41,42^

The photophysical processes in the samples were also characterized by photoluminescence (PL) at 375 nm excitation to understand the dynamic photoexcitation processes. It is interesting to note that the UiO-66-NH_2_ with and without Ru only shows a main broad PL peak but with no vibrational or progression feature, whereas UiO-67-bpydc gives a clear shoulder peak at about 550 nm (triplet state from LLCT) matching with its visible absorption regime. The PL of Ru/bpy and UiO-67-bpydc can be deconvoluted into two Gaussian peaks to quantify the peak shoulder (Figure 3c), with the fitting parameters available in Table S8. Similar to the UV–vis spectrum, there is a further broadening of the PL shoulder in Ru/bpy to capture the most visible regime, presumably to the availability of the additional lower energy triplet intermediate state due to MLCT in the metal–bipyridinic system.^43^

Time-resolved photoluminescence (TRPL) was also employed at the energy of PL peak to examine the kinetics of such charge transfer processes (Figure S11), and the data are fitted against a biexponential decay function (Table S9). For the extensive conjugation in bipyridine without Ru, the dominant charge transfer process is shown to be the higher-energy LLCT, as stated. The introduction of Ru offers another lower energy pathway that would quench the LLCT transition. Notice that the average lifetime of Ru/bpy was calculated to be 0.110 ns, which is significantly shorter compared to 0.450 ns of UiO-67-bpydc.^44^ Such sharp attenuation in exciton lifetime suggests that an relaxation pathway is via the MLCT process, which is an alternative to the radiative recombination of LLCT. Alternatively, for the isolated benzyl amine systems, it is acceptable that the high energetic process involves transfer of lone pair electrons in N to the linker molecule (n → π*, see TDDFT calculations below), the Ru could thus facilitate the polarization by further coupling with its orbitals, hence prolonging the average lifetime of UiO-66-NH_2_ and from 0.668 to 1.696 ns in the case of Ru/NH_2_. Thus, Ru is thus shown to suppress the rate of charge recombination for increased lifetime of charge carriers in this case.^45^ To further understand and evaluate the charge carrier dynamics, transient absorption spectroscopy (TAS) was performed on the Ru/bpy sample (Figure S12). Intriguingly, a positive peak at 423 nm can be observed, which is close to the PL peak determined at 426 nm. This is indicative of excited state absorption (ESA) on top of the radiative process probed by PL, echoing our proposed model for Ru/bpy. Both the broadening of the PL peaks and the ESA suggest there are various available triplet energy intermediate states due to MLCT in the Ru/bpy system, whose lifetime peaks at 2 ps.

## Excited-State Calculations

**Figure 4:**
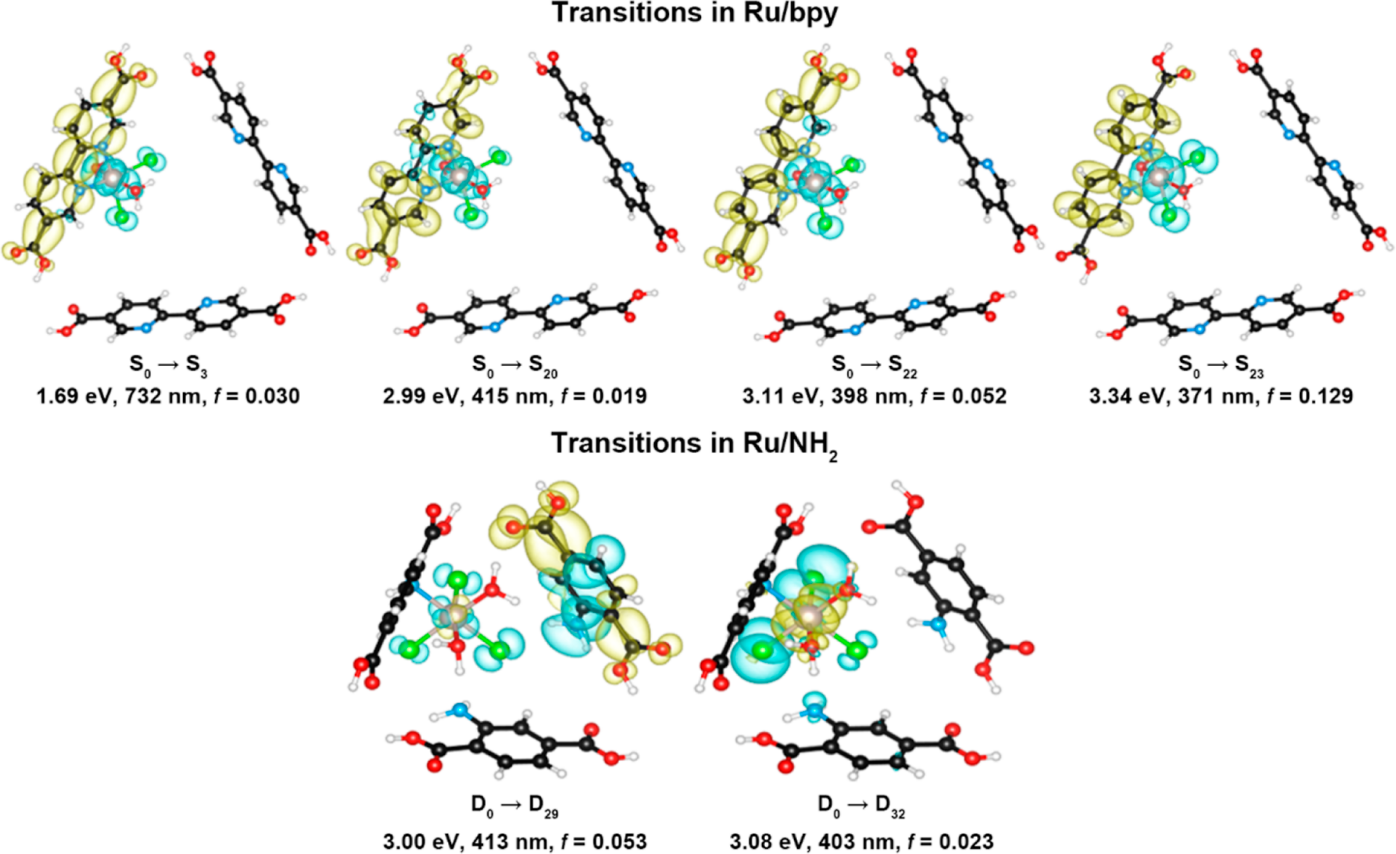
Electron–hole distributions (isosurface: 0.001 e/Å^3^) of the electronic transitions with oscillator strength being larger than 0.01 in the cluster models of Ru/bpy and Ru/NH_2_ structures. S and D denote singlets and doublet, respectively. Structure color scheme: C = black, N = blue, O = red, H = white, Ru = gray, and Cl = green. Isosurface color scheme: electron = yellow, hole = blue.

From the optical characterization, the MLCT processes of the Ru/bpy catalyst have been seen by UV–vis and PL spectroscopy, while the charge transfer kinetics have indicated a decreased lifetime of Ru/bpy compared with its parent UiO-67-bpydc. To confirm the above charge transfer processes and the nature of polarization in the Ru-MOFs, excited-state calculations were performed based on TDDFT. The Ru/bpy and Ru/NH_2_ structures were constructed by anchoring the corresponding Ru species on the linkers of the UiO-67-bpydc and UiO-66-NH_2_ frameworks, respectively (Figure S13). To reduce the computational cost, the UiO-67-bpydc and UiO-66-NH_2_ frameworks were presented by their truncated clusters (Figure S14), which has been proved to be effective in previous studies.^46,47^ The calculated most stable spin states of the cluster models of Ru/bpy and Ru/NH_2_ are closed-shell singlet and doublet, respectively (Tables S10 and S11). The electronic transitions in these two clusters (with the most stable spin state) were further explored with the aid of TDDFT. The first 50 excited states were calculated for each cluster model, and those with an oscillator strength (*f*) larger than 0.01 are presented in Figure 4 and Table S12. The oscillator strength quantifies the probability of absorption or emission of electromagnetic radiation in transitions between energy levels. In the cluster of Ru/bpy, the S_0_ → S_3_ (*f* = 0.030), S_0_ → S_20_ (*f* = 0.019), S_0_ → S_22_ (*f* = 0.052), and S_0_ → S_23_ (*f* = 0.129) transitions are relatively strong. Interestingly, the S_0_ → S_3_ transition of MLCT in origin identified at 732 nm corresponds well to the experimental 690 nm peak in UV–vis. Similarly, the calculated S_0_ → S_20_ transition (415 nm) is correlated to the MLCT peak at 487 nm of UV–vis, while the energetically close S_0_ → S_22_ and S_0_ → S_23_ transitions (398 and 371 nm, respectively) are correlated to the absorption region at 339 nm of the UV–vis. Such transitions indeed feature the excited electron and hole being localized on the linker and Ru, respectively, confirming the generation of the photoinduced but transient Ru–N Lewis acid–base pair as the dominant transition under illumination. It is well-known that excited states, in particular charge-transfer excited states, are challenging to model accurately.^48–51^ In the framework of TDDFT, the mean absolute error of electronic transition energies calculated using the decent B3LYP functional^52^ (which is also the functional used in the present study) is approximately 0.5 eV, as reported in previous benchmark studies.^53,54^ Therefore, in state-of-the-art TDDFT, the obtained transition energies agree well with the experimental results.

On the other hand, the absence of low energy available π overlap between Ru–N in Ru/NH_2_, with isolated benzyl amine and poor mixing with Ru orbitals, is evident; there are only two relatively strong electronic transitions, i.e., D_0_ → D_29_ (*f* = 0.053) and D_0_ → D_32_ (*f* = 0.023). The D_0_ → D_29_ transition at 413 nm is dominated by the electron from lone pair n in the N → aromatic π* configuration, with minimal involvement with Ru metal, which agrees well with the experimentally observed absorption peak at 371 nm in the spectrum of the Ru/NH_2_. The peak may also have the contribution from the D_0_ → D_32_ transition, which is generally localized within the Ru complex, reinforcing the absence of charge transfer between Ru and the linker (no polarization).

## Conclusions

In conclusion, a photoinduced frustrated Lewis pair like active site is, for the first time, reported in the Ru/bpy catalyst as a proof of concept. Its design combines the high chemical tunability of the MOF and the strong metal-to-ligand charge transfer ability of ruthenium complexes. The successful anchoring of Ru onto MOFs is shown with EXAFS and Rietveld refinement on SXRD data, while the optical processes are characterized by UV–vis, PL, and TRPL. Upon light irradiation, the Ru–N Lewis acid–base pair in Ru/bpy is polarized as indicated by the TDDFT calculations, beneficial to the photo catalysis of the hydrogen–deuterium gas exchange reaction. We believe our approach for catalyst design can become transferable to the wider application of light-induced FLP-like chemistries in the activation of small molecules.

## Experimental Section

Full details of the experiments can be found in the Supporting Information.

### Sample Preparation

In a 20 mL scintillation vial, 0.150 mmol ZrCl_4_ and 0.138 mmol 2-aminoterephthalic acid or 0.138 mmol 2,2-bipyridine-4,4′-dicarboxylic acid for UiO-66-NH_2_ and UiO-67-bpy, respectively, and 0.7 mL of ethanoic acid were added into 10 mL of DMF. The reaction mixture was sonicated for 10 min and then heated in an oven at 120 °C for 24 h. The formed UiO-66-NH_2_ or UiO-67-bpydc powder was collected by centrifugation at 5000 rpm for 10 min, washed by DMF three times and subsequently soaked in ethanol for 24 h three times. The products were dried overnight in a vacuum oven at 80 °C. For the synthesis of Ru/bpy and Ru/NH_2_, 200 mg of dried MOF was put into activation at 120 °C overnight and then added into 20 mL of ethanol. 107.6 mg of RuCl_3_ was then added to the UiO-67-bpydc (123.9 mg of RuCl_3_ for UiO-66-NH_2_) solution under stirring at room temperature for 24 h. The product was collected by centrifugation at 5000 rpm for 10 min, washed with ethanol 3 times. The products were dried overnight in a vacuum oven at 80 °C.

### FT-IR Measurements

FTIR experiments were performed in the range of 650–4000 cm^–1^ on a Nicolet iS 50 FT-IR spectrometer using an MCT/A detector at a resolution of 4 cm^–1^. Approximately 50 mg of Ru/bpy samples was placed in the reaction cell in the glovebox.

### SS-NMR Measurements

^1^H MAS MAS NMR experiments were conducted on a Bruker Avance 400 spectrometer operating at 9.05 T with a 4 mm double-resonance MAS probe, with Larmor frequencies of 495.43 MHz for ^1^H. Spinning speeds of 12 kHz, 32 scans, and 3.9 μs π/2 excitation pulse were used for ^1^H single-pulse acquisitions. The standard two-dimensional (2D) three-pulse exchange (a.k.a. NOESY) sequence was used to monitor the chemical exchange between proton sites in the catalyst, with a dwell time in the indirect dimension set to 20 μs. Typically, 32 scans were acquired for each *t*1 increment, with final data sets consisting of 512 *t*1 × 2048 *t*2. Mixing times is 10 ms. The recycle delays used in the 2D exchange experiments is 5 s with MAS speeds equal to 12 kHz. ^1^H chemical shift is referenced to adamantane at 1.78 ppm.

### Theoretical Calculations

The periodic structures were optimized by performing spin-polarized density functional theory (DFT) calculations using the Vienna Ab initio Simulation Package (VASP).^55–57^ The PBEsol^58^ exchange–correlation functional with the D3 dispersion correction of Grimme^59^ was applied for geometry optimizations. The core–valence electron interactions were treated by using the projector augmented wave (PAW)^60^ method. A kinetic energy cutoff of 500 eV was used for all periodic DFT calculations. A 1 × 1 × 1 *k*-point mesh was used for the sampling of the first Brillouin zone. Both atomic positions and the shape of the cell were allowed to relax during optimizations, for which we used a Hellman–Feynman force criterion of 0.05 eV/Å for each ion.

The cluster models of the Ru/NH_2_ and Ru/bpy structures were constructed by capping the [RuCl_3_(H_2_O)_2_(BDC-NH_2_)_3_]^6–^ and [RuCl_2_(H_2_O)_2_(BPYDC)_3_]^6–^ anions (from the optimized periodic models shown in Figure S11) with protons, respectively (Figure S12). The ground-state structures were obtained by a two-step optimization procedure. In the first step, only the proton capping ions were optimized. In the second step, the COOH groups of the carboxylates were fixed to retain periodic constraints, while the other atoms were allowed to fully relax. Time-dependent density functional theory (TDDFT) with the adiabatic linear-response approximation^61^ was used for excited-state calculations. The cluster calculations were performed using the Gaussian 16 program.^62^ The B3LYP^52^ exchange–correlation functional and the def2-TZVP^63–65^ basis set were used for the calculations; for Ru, effective core potential (ECP) was used. Note that B3LYP is the most commonly used functional for Ru-complexes in the reported studies.^66–69^ The electron–hole distributions were calculated by using Multiwfn^70,71^ and visualized by using VESTA.^72^

## Supporting Information Available

Additional experimental details, materials, and methods (PDF)
