# Supplementary material for: Photo-Induced Active Lewis Acid–Base Pairs in a Metal–Organic Framework for H2 Activation
Source: J Am Chem Soc. 2023 Aug 24;145(35):19312–20. doi: 10.1021/jacs.3c05244 (PMC10485891; doi:10.1021/jacs.3c05244)
Supplement: Supplementary file 1 — ja3c05244_si_001.pdf [file ja3c05244_si_001.pdf]

# Photo-Induced Active Lewis Acid-Base Pairs in Metal-Organic Framework for $H_2$ Activation

## Supporting information –
## Additional experimental details, materials, and methods


**Bryan Kit Yue Ng** – Department of Chemistry, University of Oxford, Oxford, OX1 3QR, United Kingdom

**Zi-Jian Zhou** – Key Laboratory for Advanced Materials, Centre for Computational Chemistry and Research Institute of Industrial Catalysis, East China University of Science and Technology, Shanghai 200237, People's Republic of China

**Ting-Ting Liu** – Key Laboratory for Advanced Materials, Centre for Computational Chemistry and Research Institute of Industrial Catalysis, East China University of Science and Technology, Shanghai 200237, People's Republic of China

**Tatchamapan Yoskamtorn** – Department of Chemistry, University of Oxford, Oxford, OX1 3QR, United Kingdom

**Guangchao Li** – Department of Applied Physics, The Hong Kong Polytechnic University, Hung Hom, Kowloon, Hong Kong, China

**Tai-sing Wu** – National Synchrotron Radiation Research Center, 101 Hsin-Ann Road, Hsinchu 30076, Taiwan

**Yun-Liang Soo** – Department of Physics, National Tsing Hua University, Hsinchu 30013, Taiwan

**Xin-Ping Wu*** – Key Laboratory for Advanced Materials, Centre for Computational Chemistry and Research Institute of Industrial Catalysis, East China University of Science and Technology, Shanghai 200237, People's Republic of China

**Email:** xpwu@ecust.edu.cn

**Shik Chi Edman Tsang*** – Department of Chemistry, University of Oxford, Oxford, OX1 3QR, United Kingdom

**Email:** edman.tsang@chem.ox.ac.uk

# Contents

Materials and Equipment ....4
X-ray absorption spectroscopy (XAS) measurements ....4
High throughput X-ray diffraction (XRD) measurements ....4
Inductively Coupled Plasma-Mass Spectrometry (ICP-MS) measurements....4
Ultraviolet-Visible (UV-Vis) spectroscopy measurements....5
Photoluminescence (PL) measurements ....5
Time-resolved photoluminescence (TRPL) measurements ....5
Transient Absorption Spectroscopy (TAS) measurements ....5
Photocatalytic activity measurement....5
Table S1: Amount of Ru determined by ICP-MS analysis ....6
Fig. S1 $k^3$-weighted EXAFS data and fit for (a) Ru/bpy and (b) $Ru/NH_2$ ....6
Table S2a: EXAFS fitting parameters of Ru/bpy....6
Table S2b: EXAFS fitting parameters of $Ru/NH_2$....6
Fig. S2 Comparison of scattering paths generated by Ru-N, Ru-O, Ru-Cl and Ru-Ru. ....7
Fig. S3 High-throughput X-ray diffraction of (a) Ru/bpy and UiO-67-bpydc, (b) $Ru/NH_2$ and UiO-66-$NH_2$, (c) Rietveld refinement of Ru/bpy and (d) Rietveld refinement of $Ru/NH_2$. ....7
Table S3 Atomic parameters from Rietveld refinement of SXRD measurement for Ru/bpy and $Ru/NH_2$ ....8
Fig. S4: Calibration curve of QMS signal with HD gas ....10
Fig. S5: Arrheniusm plot of Ru/bpy illuminated of the natural log of catalytic activity against the inverse of temperature ....10
Fig. S6: Full FTIR spectra of Ru/bpy illuminated and in dark....11
Fig. S7: $^1$H NMR spectra of Ru/bpy illuminated and in dark ....11
Table S4: Fitting of NMR spectrum of Ru/bpy in dark by a mixture of Gaussian/Lorentz peaks ....11
Fig. S8: Fitted $^1$H NMR spectra of Ru/bpy illuminated....12
Table S5: Fitting of NMR spectrum of Ru/bpy in dark by a mixture of Gaussian/Lorentz peaks ....12
Fig. S9: Thermogravimetric analysis (TGA) of UiO-67-bpydc....13
Table S6: Calculation of Linker to Zr ratio based on TGA data....13
Table S7: Ratio of $N(H^+)$ proton on Ru-N pair relative to bipyridinic linker protons....13
Fig. S10 UV-Vis spectra of UiO-67-bpydc and 2,2'-bipyridine-5,5'-dicarboxylic acid ....14
Table S8 Gaussian fitting parameters of PL of Ru/bpy and UiO-67-bpydc ....14
Fig. S11 TRPL of UiO-67-bpydc, Ru/bpy, UiO-66-NH2, and Ru/NH2 ....14
Table S9 TRPL fitting parameters ....15
Fig. S12 Transient Absorption Spectroscopy (TAS) of Ru/bpy....15

Fig. S13: Optimized structures of (a) the UiO-66-$NH_2$ and (b) UiO-67-bpydc frameworks with Ru species anchored on the linkers ........................................................................................16

Fig. S14: Cluster models of (a) the Ru/$NH_2$ and (b) Ru/bpy structures ..................................17

Table S10: Relative energies ($\Delta E$) of the cluster model of the Ru/bpy with various spin states ........................................................................................................................................17

Table S11: Relative energies ($\Delta E$) of the cluster model of the Ru/$NH_2$ with various spin states ........................................................................................................................................17

Table S12: Electronic transitions with oscillator strength being larger than 0.01 in the cluster models of Ru/bpy and Ru/$NH_2$ structures..........................................................................18

Scheme S1: Oxidative (blue) and reductive (red) quenching cycle of PC ………………...19

Figure S13: Amount of crotonaldehyde reduced with Ru/bpy with and without light..20

References……………………………………………………………………………………………………… … 21

# Materials and Equipment

All chemicals were obtained from commercial sources and used as received. $ZrCl_4$ (Alfa Aesar, 98%, cont. 1-2% halfnium (IV) chloride), 2,2'-Bipyridine-5,5'-dicarboxylic acid (Sigma-Aldrich, 97%), 2-aminoterephthalic acid (Merck, 99%), Ruthenium (III) chloride hydrate (Merck, 99.98%).

## X-ray absorption spectroscopy (XAS) measurements

X-ray absorption spectroscopy (XAS) data is measured at beamline BL07A of Taiwan Light Source at National Synchrotron Radiation Research Centre (NSRRC) in Taiwan. Fluorescence mode was used for Ru K-edge measurements and achieved by using a silicon drift detector. To ascertain the reproducibility of the experimental data, at least 3 scan sets were collected and compared for each sample. The EXAFS data analysis was performed using IFEFFIT with Horae packages (Athena and Artemis). The spectra were calibrated with Ru metal foil as a reference to avoid energy shifts of the samples. And the amplitude reducing parameter was obtained from EXAFS data analysis of the Ru foil, which was used as a fixed input parameter in the data fitting to allow the refinement in the coordination number of the absorption element. In this work, the first shell data analyses under the assumption of single scattering were performed with the errors estimated by R-factor[1]

## High throughput X-ray diffraction (XRD) measurements

For UiO-67-bpydc and Ru/bpy, SXRD measurements were collected at beamline BL02B2 at SPring-8, Japan. The energy of the incident X-ray flux was set at 18 keV2. The wavelength ($\lambda$ = 0.689556(2) Å) and the 2θ zero-point (ZP = -0.000015(2)°) were calibrated using a diffraction pattern obtained from a high quality $CeO_2$ powder (NIST SRM674b). High-throughput SXRD data were obtained from the MOF samples (loaded in 0.5-mm borosilicate capillaries)) using the MYTHEN detector. The patterns were collected in the 2θ range 2-78° with 0.006° data binning. Each SXRD pattern was collected for 5 min for each MYTHEN-2θstep, i.e., 10 min in total for MYTHEN data summation. This produced patterns with a good signal-to-noise ratio (S/N). Therefore, the quality of the Rietveld refinement should be best judged by the difference between the fitted and observed data[2]. For UiO-66-$NH_2$ and Ru/$NH_2$, pre-treated samples were finely sieved and loaded in 0.5-mm-borosilicate capillaries to reduce the Xray absorption problem. A high-energy X-ray using Mo anode was used to optimize the spatial and angular resolution of Bragg's reflections. The patterns were collected in the 2θ range 2-30° with 0.001° data binning. Each powder XRD pattern required 3 hours of scanning time for a suitable and reliable signal-to-noise ratio.

## Inductively Coupled Plasma-Mass Spectrometry (ICP-MS) measurements

ICP-MS data was performed on Perkin Elmer NexION 2000B ICP-MS. The calibrations were obtained using external calibration analysis with a series of standards of known concentrations prepared to form a calibration curve. The calibration standard was obtained from 10ppm standards. QM mix was used to validate the calibration curve. Standards and samples were spiked with 1ng/g Rh, In, Ir, Re so that any general instrument drift could be normalised. Dilutions were made using a 2% $HNO_3$ solution, prepared using in-house distilled nitric acid and 18.2 MΩ DI water. All data results are reported as elemental concentrations i.e. eventhough only one type of isotope is measured, the concentration values quoted are for the element inclusive of all isotopes. In the case of oxides, the values quoted are also for the element only, not the oxide of the element.

## Ultraviolet-Visible (UV-Vis) spectroscopy measurements

UV-Vis measurements was performed using Shimadzu UV-2600 under diffuse reflectance mode, with barium sulphate pallet used as the white diffuse standard. The measurement is performed from 200 to 900 nm, with a sampling interval of 0.5 nm at 298K. The slit width of the measurement is set at 5.0 nm and the wavelength of light source change from W halogen lamp to deuterium lamp was set to be 370.

## Photoluminescence (PL) measurements

PL data were recorded on Shimadzu RF-6000 spectrofluorophotometer. The excitation wavelength is 375 nm with emission wavelength monitored from 400 nm to 700nm. Data interval was set to be 0.2 nm at a scan rate of 200 nm / min. The bandwidth of excitation and emission are set to be 10 nm. Samples were prepared at a concentration of 1mg/mL with deionised water as solvent at 298K.

## Time-resolved photoluminescence (TRPL) measurements

TRPL was performed to measure the photoluminescence spectra and corresponding exciton lifetimes. A bespoke micro photoluminescence setup was used, in which a Ti-Sapphire laser (λ = 375 nm, pulse duration = 150 fs, repetition rate = 76 MHz) was directed onto the sample. Time-resolved measurements were performed using the spectrometer as a monochromator before passing the selected signal to a photomultiplier tube (PMT) detector with an instrument response function width of ~150 ps connected to a time-correlated single-photon counting module. The exciton lifetime is obtained by fitting the corresponding background-corrected TRPL spectra with a bi-exponential decay function in the form of $y = A_1e^{-x/t1} + A_2e^{-x/t2}$. Errors in the fitting were determined using a least square method.

## Transient Absorption Spectroscopy (TAS) measurements

TAS was performed on an Ultrafast Systems Helios Transient Absorption Spectrometer. Ru/bpy sample was dispersed on ethane-1,2-diol to reduce the rate of sedimentation and effect of scattering.

## Photocatalytic activity measurement

25mg of catalyst and a magnetic stirrer bar were added into an autoclave. Stirred at 500rpm, the autoclave was heated to the desired temperature before the light is shone onto the sample for 1.5 hours. Upon cool down, the autoclave was connected to the quadrupole mass spectrometer (QMS). The measurement was taken in bar mode with Faraday detector, at sensitivity from $10^{-7}$ to $10^{-12}$ Pa. For each scan, the signal from 300 scans is averaged to produce a reliable result. Mass to charge ratio of 3 has been interpreted as signal from hydrogen deuteride[3].

## Table S1: Amount of Ru determined by ICP-MS analysis

| Sample | Ru (ppm) | Zr (ppm) | Ru:Zr |
|---|---|---|---|
| $Ru/NH_2$ | 90.69 | 257.94 | 0.352 |
| Ru/bpy | 117.74 | 342.46 | 0.344 |

## Fig. S1 $k^3$-weighted EXAFS data and fit for (a) Ru/bpy and (b) $Ru/NH_2$

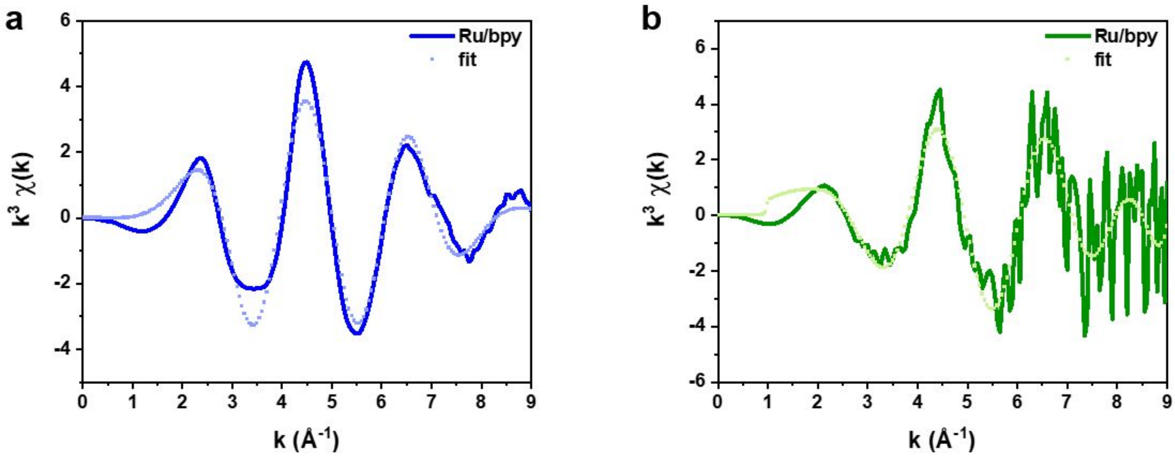


## Table S2a: EXAFS fitting parameters of Ru/bpy

| Path | CN | R / Å | D-W factor ($\sigma^2$) |
|---|---|---|---|
| Ru-N | 2.0(2) | 2.30(2) | 0.003(2) |
| Ru-O | 2.2(3) | 1.94(1) | 0.003(2) |
| Ru-Cl | 2.2(2) | 2.28(2) | 0.013(3) |

Fitting was performed with all $k^1$, $k^2$, and $k^3$-weighted R-space data. $S_0^2$ was fixed as 0.85[4] and $\Delta E_0$ was evaluated as -4.18 (37) eV. Data ranges $2 \le k \le 9$ Å$^{-1}$, $1.0 \le R \le 2.2$ Å. R factor for this fit is 0.59% (R-factor by k-weight: $k^1$: 0.54%, $k^2$: 0.41%, $k^3$:0.81%).

## Table S2b: EXAFS fitting parameters of $Ru/NH_2$

| Path | CN | R / Å | D-W factor ($\sigma^2$) |
|---|---|---|---|
| Ru-N | 0.9(1) | 1.78(4) | 0.012(2) |
| Ru-O | 1.9(1) | 2.00(1) | 0.001(1) |
| Ru-Cl 1 | 2.0(1) | 2.24(1) | 0.009(1) |
| Ru-Cl 2 | 1.0(2) | 2.43(2) | 0.015(3) |

Fitting was performed with all $k^1$, $k^2$, and $k^3$-weighted R-space data. $S_0^2$ was fixed as 0.85 and $\Delta E_0$ was evaluated as -7.53(17) eV. Data ranges $2 \le k \le 9$ Å$^{-1}$, $1.1 \le R \le 2.2$ Å with Rbkg set at 1.1Å. R factor for this fit is 0.04% (R-factor by k-weight: $k^1$: 0.02%, $k^2$: 0.03%, $k^3$:0.06%).

## Fig. S2 Comparison of scattering paths generated by Ru-N, Ru-O, Ru-Cl and Ru-Ru.

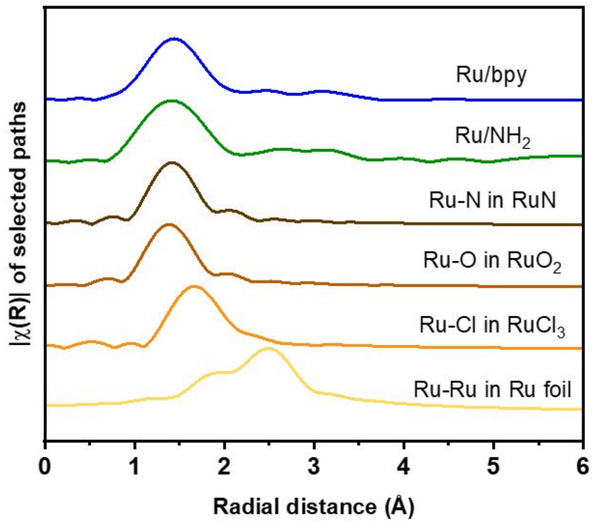


|χ(R)| denotes the magnitude of the Fourier transformed k-space data

## Fig. S3 High-throughput X-ray diffraction of (a) Ru/bpy and UiO-67-bpydc, (b) Ru/$NH_2$ and UiO-66-$NH_2$, (c) Rietveld refinement of Ru/bpy and (d) Rietveld refinement of Ru/$NH_2$.

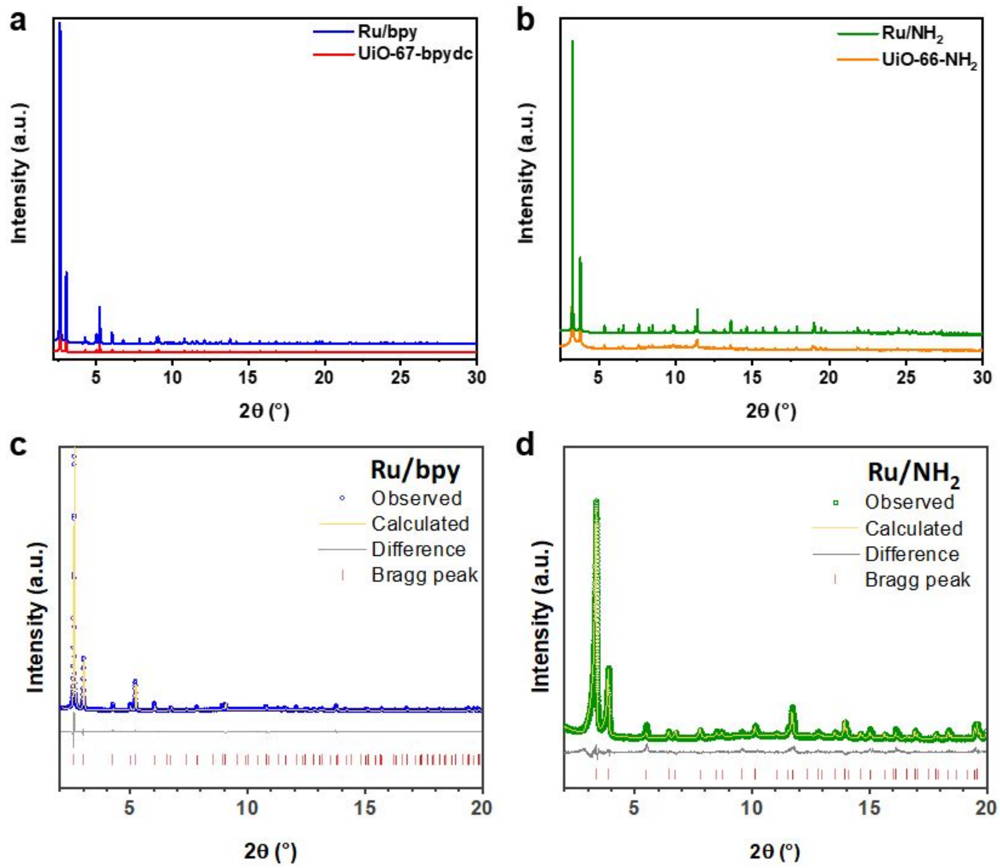

## Table S3 Atomic parameters from Rietveld refinement of SXRD measurement for Ru/bpy and Ru/$NH_2$

Unit cell of Ru/bpy, Crystal system: Cubic, Space group: Fm-3m

Unit cell parameter: a=b=c=26.5471(5) Å

Unit cell angle: α=β=γ=90°; $R_{wp}/R_p/R_{exp}$(%): 15.46/10.93/0.75. Goodness of fit: 20.52

| Atom | Multiplicity | Fractional coordinates | | | Occupancy | $B_{iso}$ (Å$^2$) |
|---|---|---|---|---|---|---|
| | | X | Y | z | | |
| Zr1 | 24 | 0.5 | 0 | 0.0932 | 1 | 0.75117 |
| O1 | 96 | 0.42693 | 0 | 0.13363 | 1 | 0.4992 |
| O2A | 32 | 0.4465 | -0.0535 | 0.0535 | 0.5 | 0.0(9) |
| O2B | 32 | 0.4594 | -0.0406 | 0.0406 | 0.5 | 0.0(9) |
| C4 | 96 | 0.3814972 | -1.916582e-16 | 0.1180148 | 0.56664 | 3.55441 |
| C5 | 96 | 0.3417866 | -9.993049e-17 | 0.1577254 | 0.56664 | 3.55441 |
| C8 | 96 | 0.2700049 | 6.40707e17 | 0.2295072 | 0.46132 | 9.92876 |
| C9 | 192 | 0.255576 | 0.002400547 | 0.1810787 | 0.23066 | 9.92876 |
| C10 | 192 | 0.292159 | 0.002373526 | 0.1452033 | 0.46132 | 9.92876 |
| N9 | 192 | 0.255576 | 0.002400547 | 0.1810787 | 0.23066 | 9.92876 |
| H1 | 32 | 0.4284 | -0.0716 | 0.0716 | 0.5 | 0.0(11) |
| H9 | 192 | 0.2209608 | 0.004061182 | 0.172211 | 0.23066 | 11.91451 |
| H10 | 192 | 0.2828014 | 0.004049805 | 0.1106683 | 0.46132 | 11.91451 |
| Ru1 | 192 | 0.18264 | 0.00488 | 0.1894 | 0.04169 | 19.96562 |
| Cl1 | 192 | 0.09744389 | 0.007884507 | 0.19611037 | 0.04169 | 19.96562 |
| Cl2 | 192 | 0.183064 | 0.008137123 | 0.1039509 | 0.04169 | 19.96562 |
| Or1 | 192 | 0.1796612 | -0.07266422 | 0.1894106 | 0.04169 | 19.96562 |
| Or2 | 192 | 0.1856311 | 0.08209076 | 0.1822214 | 0.04169 | 19.96562 |
| $O_w$1 | 96 | 0.31546 | -0.07176 | 0.07176 | 0.45478 | 19.99141 |
| $O_w$2 | 32 | 0.39485 | -0.10515 | 0.10515 | 0.42006 | 19.99141 |
| $O_w$3 | 192 | 0.33443 | 0.3354 | 0.33354 | 0.29149 | 19.99141 |
| $O_w$4 | 192 | 0.60942 | 0.51622 | -0.98548 | 0.01881 | 19.99141 |

Unit cell of Ru/$NH_2$, Space group Fm-3m
Unit cell parameter: a=b=c=20.8116(1)Å
Unit cell angle: α=β=γ=90°; $R_{wp}/R_p/R_{exp}$(%):10.58/7.59/6.86. Goodness of fit: 1.54

| Atom | Multiplicity | Fractional coordinates | | | Occupancy | $B_{iso}$ (Å$^2$) |
|---|---|---|---|---|---|---|
| | | x | Y | z | | |
| Zr1 | 24 | 0.11898 | 0 | 0 | 1 | 0.29153 |
| O2A | 32 | 0.0496 | 0.0496 | 0.0496 | 0.5 | 0.01487 |
| O2B | 32 | 0.07143 | 0.07143 | 0.07143 | 0.5 | 0.01487 |
| C1 | 48 | 0.5 | 0.20254 | 0.29746 | 0.6673 | 3.37132 |
| C2 | 192 | 0.4957595 | 0.2672635 | 0.3147305 | 0.6673 | 3.37132 |
| C3 | 48 | 0.5 | 0.14971 | 0.35029 | 1 | 0.01487 |
| O1 | 96 | 0.5 | 0.14971 | 0.35029 | 1 | 0.01487 |
| N1 | 192 | 0.3043871 | -0.0106218 | 0.1110782 | 0.166825 | 3.37132 |
| Ru1 | 192 | 0.27891 | 0.0665 | 0.08978 | 0.057 | 19.7(6) |
| Cl1 | 192 | 0.348658 | 0.07751482 | 0.1710116 | 0.057 | 19.7(6) |
| Cl2 | 192 | 0.1945092 | 0.1297852 | 0.1409389 | 0.057 | 19.7(6) |
| Cl3 | 192 | 0.2218596 | 0.04596427 | 0.0008517708 | 0.057 | 19.7(6) |
| O5 | 192 | 0.24597 | -0.01671792 | 0.129756 | 0.057 | 19.7(6) |
| O6 | 192 | 0.3050694 | 0.1539578 | 0.05407054 | 0.057 | 19.7(6) |
| $O_w$1 | 32 | 0.13971 | 0.13971 | 0.13971 | 0.65396 | 6.3672 |

$B_{iso}$: Isotropic displacement factor

**Fig. S4: Calibration curve of QMS signal with HD gas**

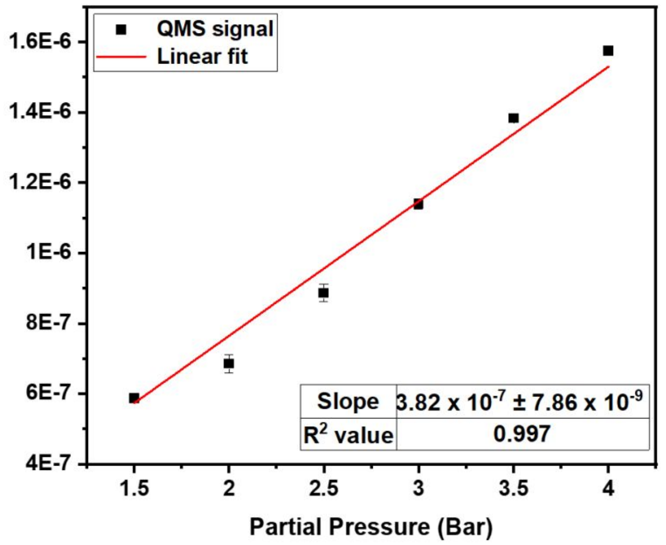


# Fig. S5: Arrheniusm plot of Ru/bpy illuminated of the natural log of catalytic activity against the inverse of temperature

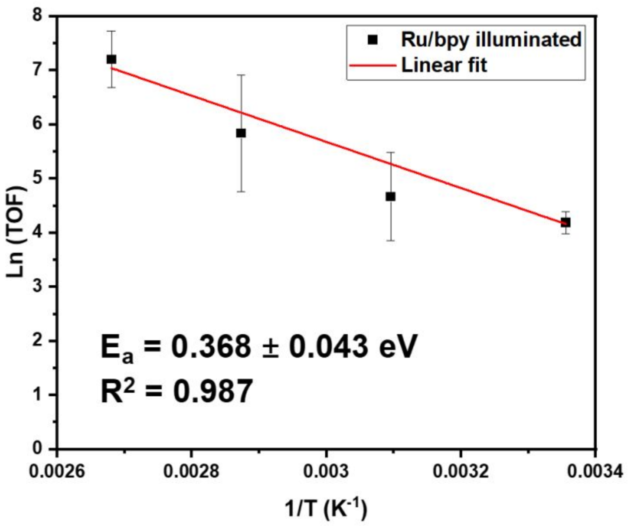

## Fig. S6: Full FTIR spectra of Ru/bpy illuminated and in dark.

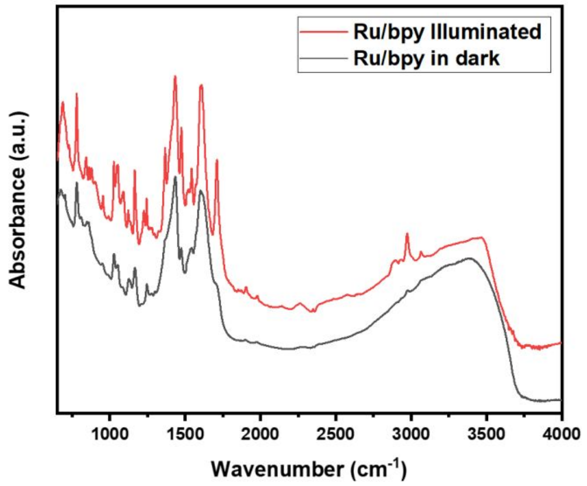


## Fig. S7: $^{1}$H NMR spectra of Ru/bpy illuminated and in dark

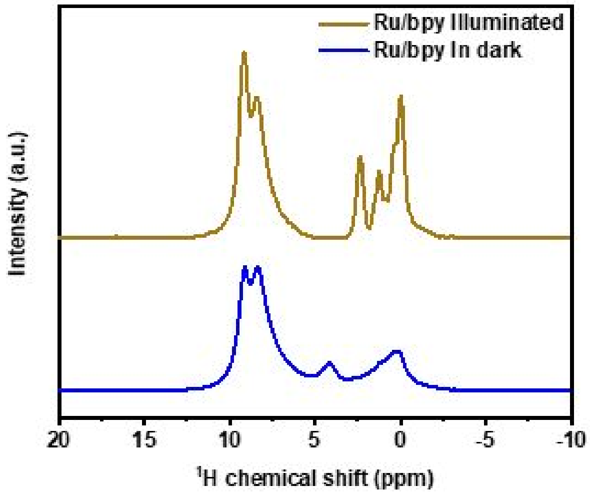


## Table S4: Fitting of NMR spectrum of Ru/bpy in dark by a mixture of Gaussian/Lorentz peaks

| Peak number | Iy ($10^8$) | Chemical shift (ppm) | LB Peak width (Hz) | % of Gaussian | Area of peak |
|---|---|---|---|---|---|
| 1 | 8.80 | 9.18 | 307 | 0.000 | 1 |
| 2 | 7.91 | 8.30 | 435 | 1.000 | 0.87 |
| 3 | 3.19 | 7.14 | 786 | 0.000 | 0.91 |
| 4 | 1.88 | 4.16 | 445 | 0.000 | 0.31 |
| 5 | 1.76 | 1.20 | 704 | 0.000 | 0.45 |
| 6 | 2.75 | 0.18 | 440 | 0.199 | 0.42 |

Best overlap (Parameter quantifying the overlap between data and fit): 94.10%
Iy: Signal intensity parameter, LB: Line broadening parameter
% of Gaussian: A full Lorentz curve has this parameter = 0, whereas a full Gaussian curve has this parameter = 1

## Fig. S8: Fitted $^1$H NMR spectra of Ru/bpy illuminated

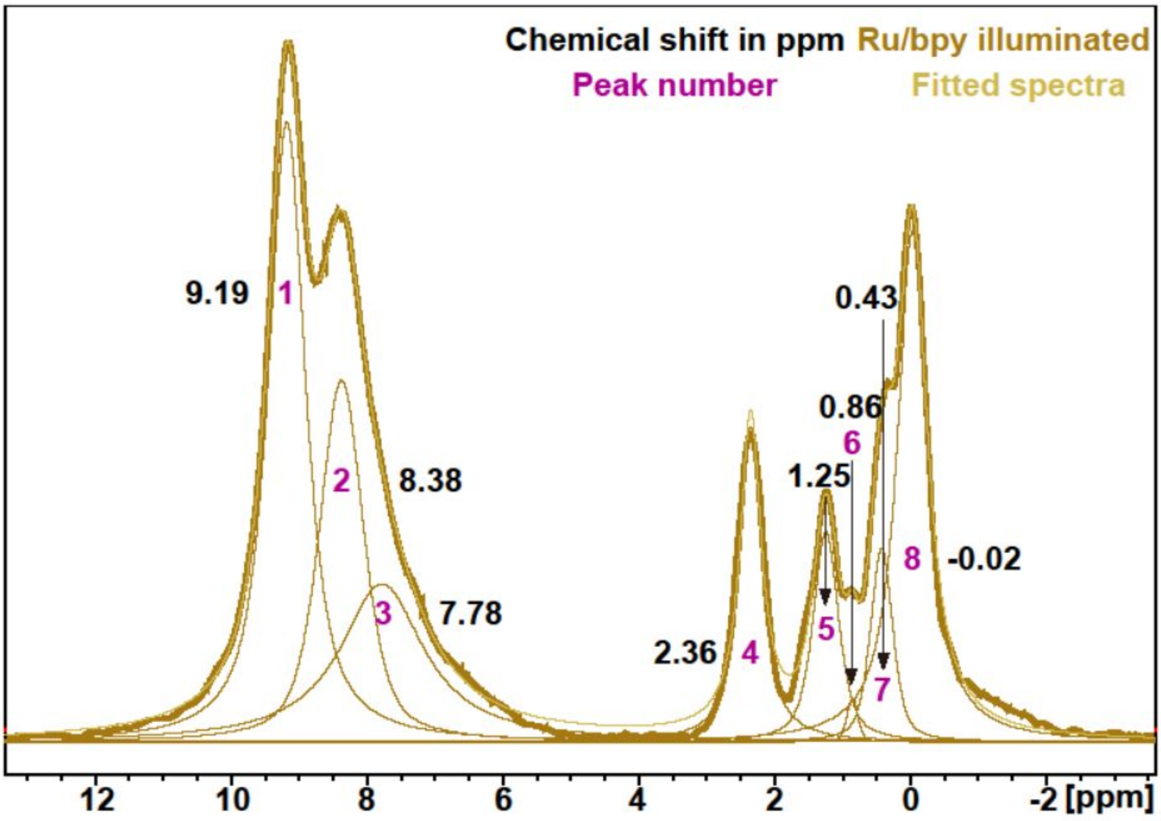


## Table S5: Fitting of NMR spectrum of Ru/bpy in dark by a mixture of Gaussian/Lorentz peaks

| Peak number | Iy ($10^8$) | Chemical shift (ppm) | LB Peak width (Hz) | % of Gaussian | Area of peak |
|---|---|---|---|---|---|
| 1 | 4.88 | 9.19 | 261 | 0.181 | 1 |
| 2 | 8.69 | 8.38 | 307 | 0.459 | 0.62 |
| 3 | 3.79 | 7.78 | 597 | 0.000 | 0.61 |
| 4 | 7.55 | 2.36 | 149 | 0.000 | 0.31 |
| 5 | 5.04 | 1.25 | 178 | 0.382 | 0.22 |
| 6 | 1.04 | 0.86 | 76.7 | 0.654 | 0.02 |
| 7 | 4.65 | 0.43 | 154 | 0.493 | 0.17 |
| 8 | 2.27 | -0.02 | 211 | 0.031 | 0.70 |

Best overlap (Parameter quantifying the overlap between data and fit): 94.62%
Iy: Signal intensity parameter, LB: Line broadening parameter
% of Gaussian: A full Lorentz curve has this parameter = 0, whereas a full Gaussian curve has this parameter = 1
Relative peak size of bipyridine (7-9 ppm) to N-H$^+$ (2.4 ppm):

$$\frac{Area\ of\ peak\ 4}{Area\ of\ peak\ 1+2+3} = \frac{0.31}{1+0.62+0.61} = \frac{1}{7.19}$$

## Fig. S9: Thermogravimetric analysis (TGA) of UiO-67-bpydc

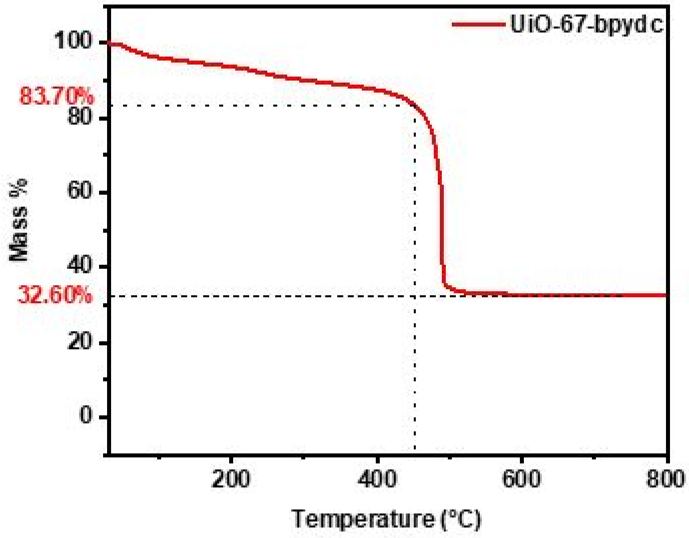


Note: Temperature at which bipyridinic linker starts to decompose is taken to be 450°C, with mass loss before that temperature attributed to adsorbed solvent[5].

## Table S6: Calculation of Linker to Zr ratio based on TGA data

| Sample | $ZrO_2$ | Linker |
|---|---|---|
| Mass (%) | 32.60 | 83.70-32.60 = 51.10 |
| Molar mass (g mol$^{-1}$) | 123.218 | 244.21 |
| Mol of Zr and linker present | 0.265 | 0.209 |
| To whole number ratio | 1 | 0.791 |

## Table S7: Ratio of N(H$^+$) proton on Ru-N pair relative to bipyridinic linker protons

| | |
|---|---|
| Ru:Zr of Ru/bpy ratio from ICP | 0.344 |
| Linker:Zr ratio from TGA | 0.791 |
| Ru/linker : linker | (0.791-0.344):0.344 = 0.447:0.344 |
| Simplest ratio | 1.30:1 |
| Available pyridinic linker to Ru-N pair ratio | 2.30:1 |
| Expected ratio of N(H$^+$) at 2.4 ppm relative to bipyridine protons* | 2.30 x 6 : 1 x 2<br>6.90:1 |

* Protons on pyridinic linker = 6
Protons expected from N(H$^+$) after charge transfer = 2

## Fig. S10 UV-Vis spectra of UiO-67-bpydc and 2,2'-bipyridine-5,5'-dicarboxylic acid

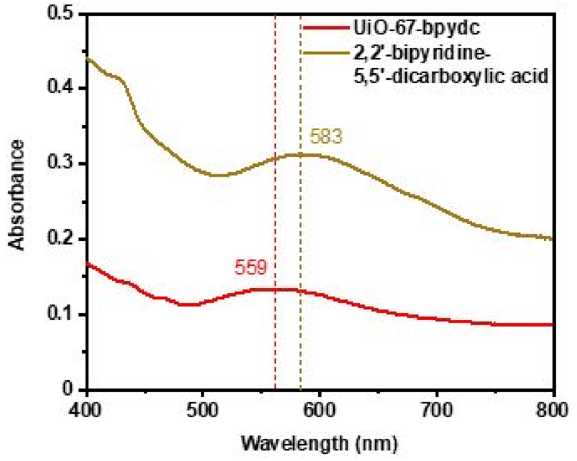


## Table S8 Gaussian fitting parameters of PL of Ru/bpy and UiO-67-bpydc

| Sample | Peak centre (nm) | Peak area ($10^6$) | Peak width |
|---|---|---|---|
| Ru/bpy peak 1 | 426.3(5) | 2.96(7) | 121(3) |
| Ru/bpy peak 2 | 544.1(19) | 0.91(4) | 105(2) |
| UiO-67-bpydc peak 1 | 419.8(6) | 5.93(17) | 110(2) |
| UiO-67-bpydc peak 2 | 522.2(34) | 1.92(13) | 121(4) |

## Fig. S11 TRPL of UiO-67-bpydc, Ru/bpy, UiO-66-NH2, and Ru/NH2

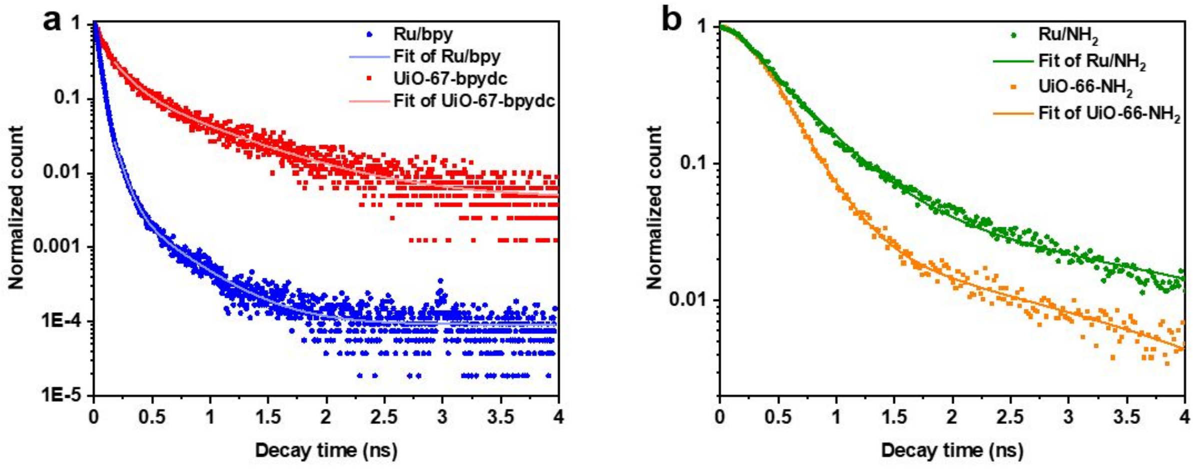

## Table S9 TRPL fitting parameters

| Samples | $A_1$ | $t_1$ | $A_2$ | $t_2$ | Average lifetime | $R^2$ value |
|---|---|---|---|---|---|---|
| UiObpy | 0.565(20) | 0.155(7) | 0.162(10) | 0.684(31) | 0.450(150) | 0.984 |
| Ru/bpy | 0.233(4) | 0.076(1) | 0.006(0) | 0.375(10) | 0.110(57) | 0.998 |
| $UiONH_2$ | 2.387(56) | 0.262(4) | 0.036(4) | 3.012(202) | 0.668(200) | 0.998 |
| $Ru/NH_2$ | 1.187(6) | 0.437(4) | 0.061(5) | 4.228(453) | 1.696(219) | 0.998 |

Average lifetime is calculated by the formula:

$$t_{average} = \frac{A_1 t_1^2 + A_2 t_2^2}{A_1 t_1 + A_2 t_2}$$

## Fig. S12 Transient Absorption Spectroscopy (TAS) of Ru/bpy

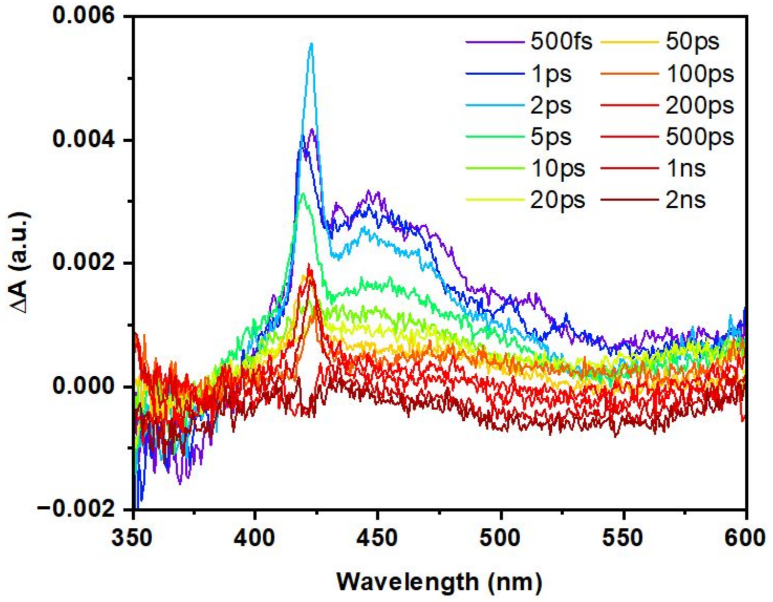

**Fig. S13: Optimized structures of (a) the UiO-66-$NH_2$ and (b) UiO-67-bpydc frameworks with Ru species anchored on the linkers**

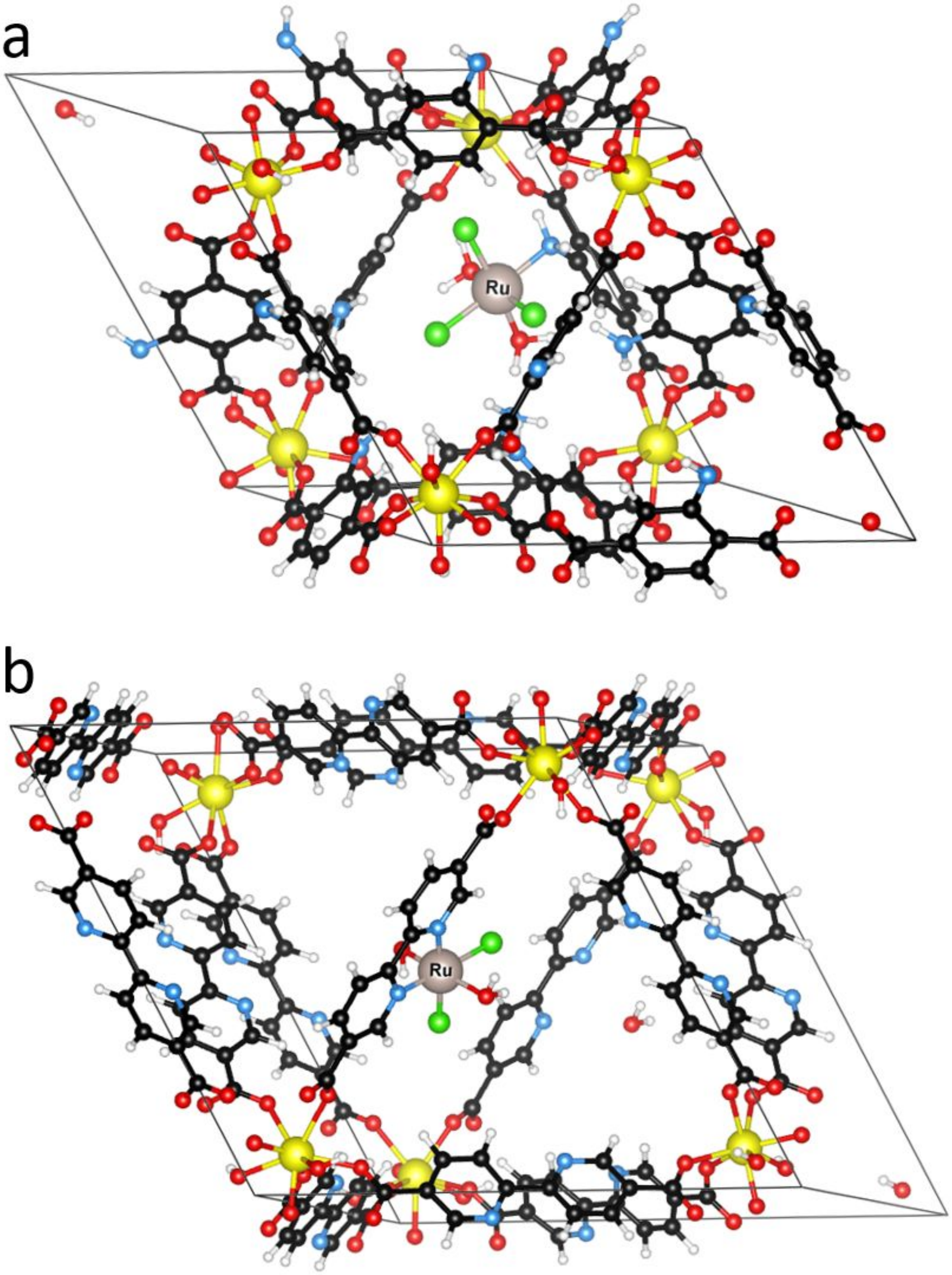

## Fig. S14: Cluster models of (a) the Ru/$NH_2$ and (b) Ru/bpy structures

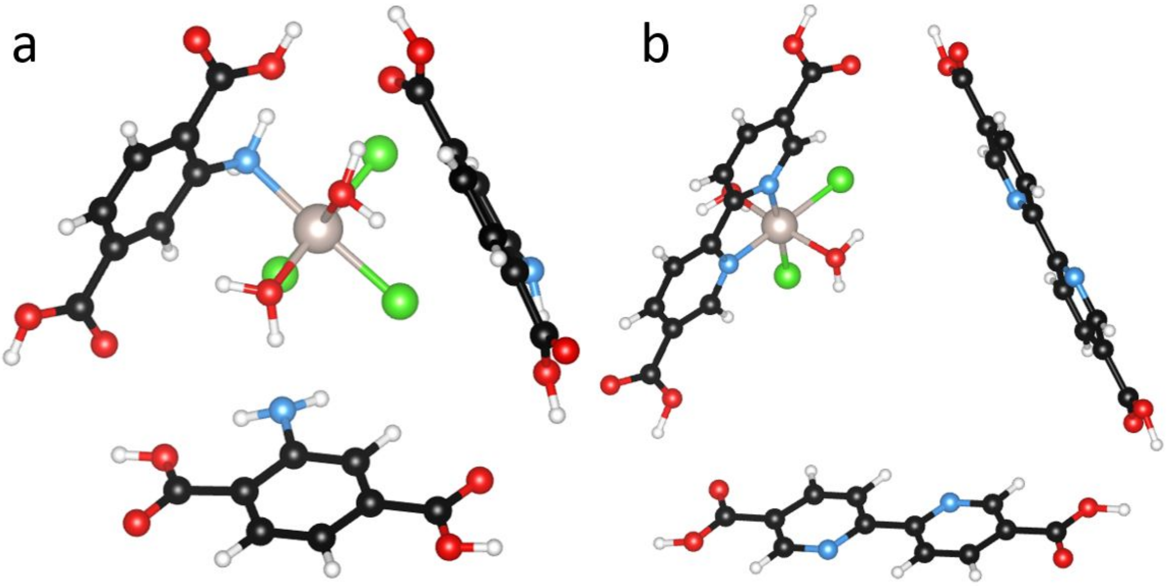


## Table S10: Relative energies ($\Delta E$) of the cluster model of the Ru/bpy with various spin states

| Multiplicity | $\Delta E$ (eV) |
|---|---|
| Singlet | 0 |
| Triplet | 0.83 |
| Quintet | 1.67 |

## Table S11: Relative energies ($\Delta E$) of the cluster model of the Ru/$NH_2$ with various spin states

| Multiplicity | $\Delta E$ (eV) |
|---|---|
| Doublet | 0 |
| Quartet | 0.74 |
| Sextet | 1.25 |

## Table S12: Electronic transitions with oscillator strength being larger than 0.01 in the cluster models of Ru/bpy and Ru/$NH_2$ structures

| Transition | Energy | Oscillator Strength, $f$ |
|---|---|---|
| Ru/bpy | | |
| $S_0 \rightarrow S_3$ | 1.69 eV, 732 nm | 0.030 |
| $S_0 \rightarrow S_{20}$ | 2.99 eV, 415 nm | 0.019 |
| $S_0 \rightarrow S_{22}$ | 3.11 eV, 398 nm | 0.052 |
| $S_0 \rightarrow S_{23}$ | 3.34 eV, 371 nm | 0.129 |
| Ru/$NH_2$ | | |
| $D_0 \rightarrow D_{29}$ | 3.00 eV, 413 nm | 0.053 |
| $D_0 \rightarrow D_{32}$ | 3.08 eV, 403 nm | 0.023 |

S and D denote singlet and doublet, respectively

## Scheme S1: Oxidative (blue) and reductive (red) quenching cycle of PC [6]

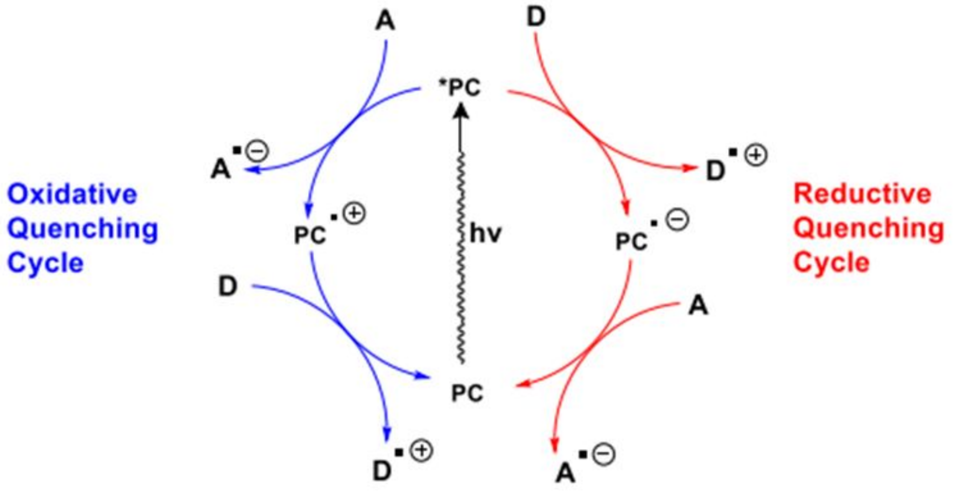


It is noted that homogeneous Ru pyridyl complexes can be used as photocatalyst (PC) which absorbs light in which substrate in the catalysis does not absorb. Upon photo-excitation, it is well established that the photocatalyst can engage in SET (single electron transfer) with electron donors (Ru) or electron acceptors (organics) since photoexcited species are both stronger oxidants and reductants than the ground state species. Consequently, excited photocatalysts can be transformed either by oxidative or reductive quenching with added redox chemical species in a separated manner (**Scheme S1**). In the reductive quenching cycle, the excited species functions as an oxidant in the SET and accepts an electron from an electron donor referred to as a reductive quencher. A reduced species $PC^{\bullet-}$ is formed in this SET process which is a reductant as well and may further donate an electron to regenerate the initial redox state[7]. Tertiary amines are commonly used as reductive quenchers[8] and common oxidative quenchers include viologens, polyhalomethanes, dinitro- and dicyanobenzenes, and aryldiazonium salts[9]. Although light-induced generation of $Ru^{+}$-pyridyl$^{-}$ through MLCT is generally concluded over such systems, as far as we are aware that there is no claim in the literature for the homogeneous $Ru(bpy)_3^{2+}$ (or other photosensitizing metal-organic complexes) to carry out heterolytic synergetic cleavage of $H_2$ via 'transit FLP' in hydrogenation reactions, let alone the direct proof of the 'excited species' in MOF isolated counterpart for the 'transient FLP' as demonstrated in our case. This is the main novelty we present in this paper over the existing literature. At this stage, we do not yet know whether it is unique for MOF stabilized Ru-bipyridine or can be expanded to homogeneous systems (Ru mono-pyridyl is subjected to rapid deactivation) where more experiments are required. However, if the excited electron is delocalized in the bipyridine centred ligand position ($\pi^*$), as shown in both experimentally and theoretically in homogeneous systems[10] which is different from our IR/NMR identified $N^-H^+$ (excited electron is localised in N) with simultaneously formation of $Ru^+$-$H^-$ in the charge synergetic $H_2$ activation by MOF stabilized Ru-bipyridine by photo FLP chemistry. With electronic coupling and mixing of electronic wave functions, the transition between localization and delocalization, the reorganization energy, the intramolecular and solvent reorganization energies with and without encapsulation in MOF framework should be carefully compared before we could

elucidate this transit FLP mechanism at later time. Thus, our novelty lays in the charge transfer as a consequence of the catalyst design being able to heterolytically activate hydrogen in a Frustrated Lewis Pair-like (FLP-like) manner. Such photo-induced charge separation from neighbouring Lewis acid and base sites without bulk group can be evident. In this system, the bipyridine group of UiO-series MOF as ligand is chelated to Ru metal ion in close proximity. Upon light activation, electron transfer can be momentarily induced from the metal to ligand to facilitate heterolytic H-H bond cleavage, with the advantage of a precise control of active site location and quantity in this solid catalyst. As a result, we consider such a report of photo-induced FLP-like activation sites for the first time.

## Figure S13: Amount of crotonaldehyde reduced with Ru/bpy with and without light.

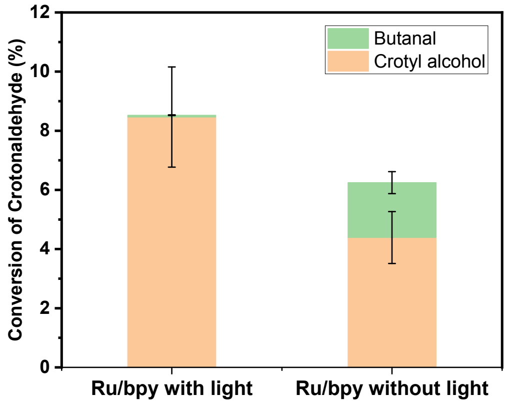


In addition, we have briefly studied the hydrogenation of crotonaldehyde as a model hydrogenation reaction at conditions similar to the hydrogen-deuterium exchange reaction we performed (100°C, 4 bars H2) at the same batch reactor as outlined in the supplementary information (Fig.S13). Notice that a promotion of catalytic reduction activity of Ru/bpy with light, similar to that in the hydrogen-deuterium exchange reaction, can be observed. Also, the charge ($H^+$ and $H^-$) induced hydrogenation are clearly more specific for hydrogenation of crotonaldehyde to corresponding unsaturated alcohol than that of radical-like hydrogenation by thermal means (higher butanal selectivity).

# References

(1) Ravel, B.; Newville, M. ATHENA, ARTEMIS, HEPHAESTUS: data analysis for X-ray absorption spectroscopy using IFEFFIT. *J Synchrotron Radiat* **2005**, *12* (Pt 4), 537-541. DOI: 10.1107/S0909049505012719.
(2) L. B. McCusker, R. B. V. D., D. E. Cox, D. Louer, P. Scardi. Rietveld refinement guildlines. *J. Appl. Cryst.* **1999**, *32*, 36-50.
(3) R. E. Ellefson, W. E. M. Hydrogen isotope analysis by quadrupole mass spectrometry. *J. Vac. Sci. Techhnol.* **1981**, *18(3)*.
(4) König, C. F. J.; van Bokhoven, J. A.; Schildhauer, T. J.; Nachtegaal, M. Quantitative Analysis of Modulated Excitation X-ray Absorption Spectra: Enhanced Precision of EXAFS Fitting. *The Journal of Physical Chemistry C* **2012**, *116* (37), 19857-19866. DOI: 10.1021/jp306022k.
(5) Xu, Z.; Zhao, G.; Ullah, L.; Wang, M.; Wang, A.; Zhang, Y.; Zhang, S. Acidic ionic liquid based UiO-67 type MOFs: a stable and efficient heterogeneous catalyst for esterification. *RSC Adv* **2018**, *8* (18), 10009-10016. DOI: 10.1039/c8ra01119b From NLM PubMed-not-MEDLINE.
(6) Medina, E.; Sandoval-Pauker, C.; Salvador, P.; Pinter, B. Mechanistic Insights into the Oxidative and Reductive Quenching Cycles of Transition Metal Photoredox Catalysts through Effective Oxidation State Analysis. *Inorg Chem* **2022**, *61* (47), 18923-18933. DOI: 10.1021/acs.inorgchem.2c02945 From NLM PubMed-not-MEDLINE.
(7) Shaw, M. H.; Twilton, J.; MacMillan, D. W. Photoredox Catalysis in Organic Chemistry. *J Org Chem* **2016**, *81* (16), 6898-6926. DOI: 10.1021/acs.joc.6b01449 From NLM PubMed-not-MEDLINE.
(8) Kim, H.; Lee, C. Visible-light-induced photocatalytic reductive transformations of organohalides. *Angew Chem Int Ed Engl* **2012**, *51* (49), 12303-12306. DOI: 10.1002/anie.201203599 From NLM PubMed-not-MEDLINE.
(9) Schroll, P.; Hari, D. P.; Konig, B. Photocatalytic arylation of alkenes, alkynes and enones with diazonium salts. *ChemistryOpen* **2012**, *1* (3), 130-133. DOI: 10.1002/open.201200011 From NLM PubMed-not-MEDLINE.
(10) Thompson, D. W.; Ito, A.; Meyer, T. J. [Ru(bpy)3]2+* and other remarkable metal-to-ligand charge transfer (MLCT) excited states. *Pure Appl Chem* **2013**, *85* (7), 1257-1305. DOI: 10.1351/pac-con-13-03-04.
